# Assessment of cortical inhibition depends on inter individual differences in the excitatory neural populations activated by transcranial magnetic stimulation

**DOI:** 10.1038/s41598-022-14271-1

**Published:** 2022-06-15

**Authors:** Andris Cerins, Daniel Corp, George Opie, Michael Do, Bridgette Speranza, Jason He, Pamela Barhoun, Ian Fuelscher, Peter Enticott, Christian Hyde

**Affiliations:** 1 Cognitive Neuroscience Unit, School of Psychology, Deakin University, Burwood Campus, 221 Burwood Hwy, Burwood, VIC 3125 Australia; 2 Center for Brain Circuit Therapeutics, Brigham and Women’s Hospital, Boston, MA USA; 3 Discipline of Physiology, School of Biomedicine, The University of Adelaide, Adelaide, SA 5005 Australia; 4 Department of Forensic and Neurodevelopmental Sciences, Sackler Institute for Translational Neurodevelopment, Institute of Psychiatry, Psychology, and Neuroscience, King’s College London, London, UK

**Keywords:** Neural circuits, Synaptic transmission

## Abstract

Transcranial magnetic stimulation (TMS) is used to probe inhibitory intracortical neurotransmission and has been used to infer the neurobiological dysfunction that may underly several neurological disorders. One technique, short-interval intracortical inhibition (SICI), indexes gamma-aminobutyric acid (GABA) mediated inhibitory activity and is a promising biomarker. However emerging evidence suggests SICI does not exclusively represent GABAergic activity because it may be influenced by inter-individual differences in the specific excitatory neural populations activated by TMS. Here we used the latency of TMS motor evoked potentials (MEPs) to index these inter-individual differences, and found that a significant proportion of the observed variability in SICI magnitude was accounted for by MEP latency, *r* = − 0.57, *r*^2^ = 0.33, *p* = .014. We conclude that SICI is influenced by inter-individual differences in the excitatory neural populations activated by TMS, reducing the precision of this GABAergic probe. Interpreting SICI measures in the context of MEP latency may facilitate a more precise assessment of GABAergic intracortical inhibition. The reduced cortical inhibition observed in some neuropathologies could be influenced by reduced activity in specific excitatory neural populations. Including MEP latency assessment in research investigating SICI in clinical groups could assist in differentiating the cortical circuits impacted by neurological disorders.

## Introduction

Short-interval intracortical inhibition (SICI) is a paired-pulse transcranial magnetic stimulation (TMS) technique that provides a non-invasive indication of intracortical inhibitory activity mediated by gamma-aminobutyric acid (GABA) A receptors^1^. SICI is abnormally reduced in several neurological disorders^2–4^, has recently informed the differentiation of dementia subtypes^5^, and has been found to predict response to TMS neuromodulation^6^. However, SICI assessment is influenced by inter-individual differences in the neural populations activated by TMS^7,8^, and this relationship may explain up to half of the inter-individual variability observed in SICI^7^. Indeed, it has been suggested that levels of SICI may depend on individual patterns of TMS neural recruitment as much as they do on the inhibitory activity that SICI attempts to assess^9^. Although SICI provides a valuable non-invasive marker of GABAergic activity, our understanding of how SICI assessment is affected by individual differences in the neural populations recruited by TMS remains incomplete.

Careful modification of TMS intensity, and cortical current direction (i.e., via changes in TMS coil orientation) can reveal inter-individual variations in the neural populations activated by TMS^10^. Invasive epidural recordings reveal that TMS delivered over the primary motor cortex (M1) elicits a descending cortico-spinal volley (CSV) of distinct waves, and individual waves are currently thought to represent activity in non-identical neural populations^11,12^. The CSV can produce a motor evoked potential (MEP) in peripheral muscle, which is quantifiable with electromyography (EMG)^13,14^. The onset latency of these MEPs can be used to indicate the likely neural populations activated in an individual^11^. Higher intensity latero-medially (LM) directed stimulation can directly activate axons of cortico-spinal neurons. Here the CSV commences with its earliest possible component, known as a direct (D) wave, and the later components, termed indirect (I) waves (I1, I2, I3 etc.), are generated by trans-synaptic activation of cortico-spinal neurons^15^. The presence of D waves in a CSV evokes MEPs of the shortest latency, which can be used to control for individual differences in the cortex to muscle pathway^16^. Lower intensity TMS delivered using posterior-anterior (PA) directed current tends to evoke a CSV commencing with an earlier I1-wave. Here, the latency of the CSV and resulting MEP is 1–1.5 ms longer than when D waves are present. Using an anterior–posterior (AP) directed current tends to evoke CSVs commencing with a later I wave (often corresponding to the I3 wave), and the resultant MEPs are approximately 3–7 ms later than D wave latency^10,17^. (See supplementary material Fig. S1 for illustration.) In summary, PA and AP MEP latencies, with LM latency subtracted, provide a non-invasive indication of the earliest component of the CSV that is recruited following TMS to M1^16^.

Crucially it is only the later I waves (I3 and later) that appear to be inhibited by SICI, any early I waves present during the test are not reduced in amplitude^18,19^. (See supplementary material Fig. S2 for illustration.) SICI uses a subthreshold (i.e., below the stimulation intensity required to elicit an MEP) conditioning stimulus (CS) to activate inhibitory interneurons that suppress the amplitude of the MEP elicited by a test stimulus (TS) delivered 1–6 ms later^20^. At a 1 ms inter-stimulus interval, the suppression is thought to be substantially influenced by the neuronal refractory period^1^. While at a 2.5–3 ms interval the extent of this reduction in MEP amplitude, compared to MEP amplitude elicited by the TS alone, indicates levels of GABAergic inhibition^1^ but may also reflect individual differences in the composition of the TMS-evoked CSV^9^. The observations that the TS inhibition stems only from inhibition of later I waves^18,21^ provide a theoretical basis for the reports^7,8^ of a relationship between MEP latency difference and SICI. Given that early I waves are not inhibited by the CS, their presence would likely contribute to TS MEP amplitude and therefore reduce the assessed SICI. Interestingly, in individuals with longer MEP latency difference, and hence no unaffected early I waves in their CSV, SICI may provide a more accurate measure of GABAergic inhibitory activity.

Two recent reports have begun to describe the practical nature of the relationship between SICI and MEP latency difference^7,8^. One reported an association between AP latency and SICI assessed in the PA direction^8^ and another reported a strong association between AP latency and AP SICI^7^. In both cases, longer MEP latency differences were associated with greater assessed inhibition, in line with what would be expected given the specific interaction each measure has with the CSV. Both of these studies utilized threshold tracked (tt) SICI where the TS intensity is increased until it overcomes the inhibitory influence of the CS^22^. Given that adjustment to stimulus intensity is known to alter CSV composition^17,23^, and that the composition of the CSV appears to drive the relationship under investigation, we therefore investigated the untested relationship between conventional amplitude-ratio SICI (where the TS intensity is held constant) measured in the PA and AP current directions and MEP latency difference. We expected that greater SICI would be associated with longer latency difference.

## Materials and methods

### Participants

Twenty-eight (9 male) healthy right-handed participants aged 18–42 (*M* = 25.22; *SD* = 5.37) were recruited from an Australian university and surrounding area via online and poster advertisements. Standard TMS exclusion criteria and pre/post safety screening procedures were applied^24,25^, including exclusion of potential participants with any self-reported history of neurological or psychological disorder, or current medical or recreational use of psychoactive drugs. The study was approved by the Deakin University Human Research Ethics Committee and all participants provided written informed consent in accordance with the Declaration of Helsinki.

### Experimental protocol

**Figure 1:**
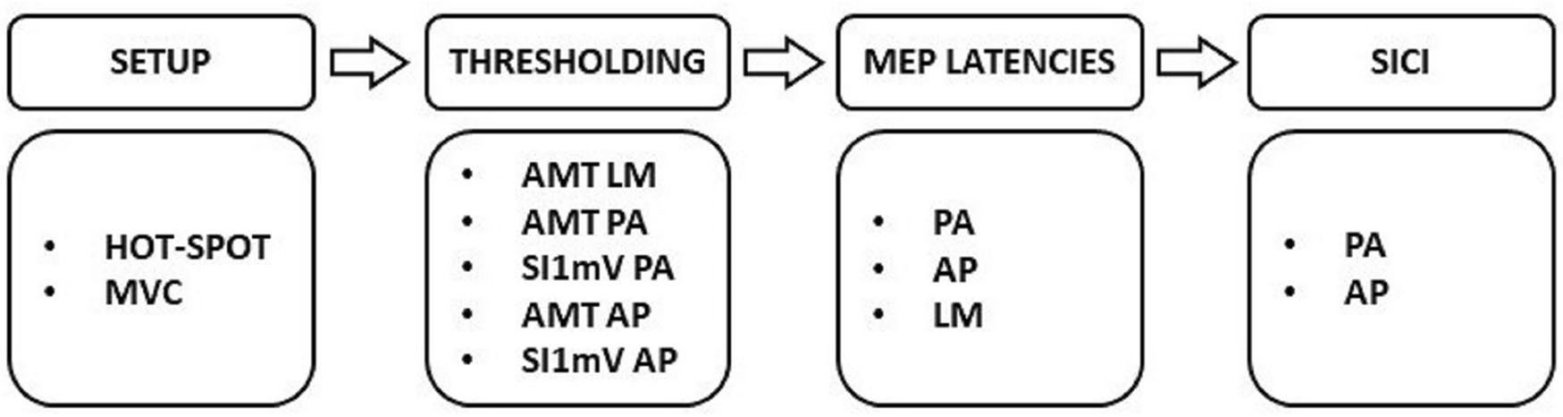
Experimental procedure. *MVC* maximum voluntary contraction, *AMT* active motor threshold, *SI* stimulus intensity, *SICI* short-interval intracortical inhibition, *PA* posterior–anterior, *AP* anterior–posterior, *LM* latero-medial.

Participants were seated in an adjustable chair with their right arm resting on a table positioned just above their lap. See Fig. 1 for the experimental procedure (described in detail below). Briefly, the experiment began with locating the stimulation site, then the relevant coil orientations were used to determine motor thresholds, apply single pulse TMS to assess MEP latency, and administer paired pulse stimulation to assess intracortical inhibition. Experiments were well tolerated by participants and no significant adverse effects were reported.

### Electromyographic recording

Surface EMG was recorded from the first dorsal interosseous (FDI) muscle of the right hand. Wet gel 10 mm Ag/AgCl electrodes were applied in a belly-tendon montage, grounded on the ulnar styloid process. EMG signals were amplified (× 1000; Bio Amp–ADInstruments New Zealand), bandpass filtered (1 Hz–2 kHz), digitized (10 kHz; PowerLab 4/35; ADInstruments), and recorded (LabChart 8.0—ADInstruments) from 200 ms before to 300 ms after TMS pulses^26^.

### Transcranial magnetic stimulation

Monophasic TMS pulses of 100 µs rise time and 1 ms duration were generated with a Magstim Bistim^2^ system (Magstim United Kingdom) and delivered with a 70 mm Magstim figure-8 coil via the Bistim connecting module. Hotspot and thresholds were assessed via single pulses delivered in Bistim mode, latency and SICI blocks were assessed in independent Bistim triggering mode^27^. The left M1 FDI hot spot for the contralateral right hand was located in accordance with previous guidelines^26^ using PA current (note, all current directions refer to cortical current). The hot spot location was marked in a neuro-navigation system (BrainSight, Rogue Research Inc. Canada) and used for all subsequent TMS. Coil position was continually monitored throughout the experiment using the neuro-navigation system. Coil position errors were low with an average (and SD) distance from the hot spot of 1.15 (0.80) mm, tilt away from the target of 2.06 (1.59) degrees and a mean absolute twist error of 1.59 (1.50) degrees. Motor thresholds were assessed with 30 TMS pulses using maximum-likelihood parameter estimation by sequential testing, implemented in the TMS Motor Assessment Tool 2.0^28^, to establish a stimulus intensity with a 50% probability of eliciting an MEP meeting the target criteria. For active motor threshold (AMT) which was assessed in PA, AP and LM current directions, the MEPs meeting criteria had amplitudes greater than 100 µV^29^, were distinguishable from background EMG, and occurred within a physiologically possible latency range (15–35 ms). During active thresholding, and subsequent latency assessments in active muscle, participants maintained a slight voluntary contraction of the FDI muscle (10% of maximum as measured with a force transducer and guided by visual feedback). Stimulus intensity with a target amplitude of 1 mV (SI1mV) was assessed in resting muscle in PA and AP current directions.

### MEP latency assessment

MEP onset was assessed as per Hamada et.al^16^ using PA, AP, and LM current directions, with intensities determined by reference to the thresholds established in each direction. For PA and AP latencies in active muscle, 20 trials were assessed in each direction using a stimulus intensity set at 110% AMT. LM latencies were assessed with 10 trials in active muscle, and a stimulus intensity of 150% AMT in order to increase the likelihood of evoking an MEP commencing with a D wave^30^. Here, as previously^16^, 10 trials at this higher stimulus intensity provided sufficiently reliable latency estimates^31^. MEP onset was also assessed in the unconditioned test pulses used to calculate SICI. These comprised 20 trials delivered at SI1mV in resting muscle, using both PA and AP current.

### SICI assessment

SICI was assessed in resting muscle in the PA and AP current directions. The CS was delivered at 90% AMT, followed 2.5 ms later by the TS delivered at SI1mV^32,33^. The intensities were determined with reference to the relevant thresholds established in each direction. Twenty SICI conditioned trials and 20 single pulse SI1mV trials were interleaved and jittered with 5, 6, and 7 s inter-stimulus intervals in pseudo-randomized order.

### Data analysis

Because voluntary contraction can substantially alter the composition of the CSV^34^, and also alter SICI^35^, any trials with EMG amplitude greater than 20 µV in the 100 ms prior to the stimulus (24%, 545 of 2240 trials) were excluded from SICI calculations. Because SICI calculation relies on averaged amplitudes, we made a pragmatic decision that any average amplitudes calculated from less than 5 MEPs were likely unreliable^36^ and therefore were not calculated. This meant that in any block of 20 SICI trials (conditioned or unconditioned) the median number of trials averaged was 18 (min 5, max20). We ultimately calculated SICI_PA_ magnitudes for 23 participants and SICI_AP_ magnitudes for 25 participants. MEP latencies were calculated for each participant and each group of latency trials using a custom Matlab script. EMG signals for the block were averaged^8^ and the latency was identified as the first timepoint following 15 ms post stimulus where the averaged signal exceeded the mean plus 5 standard deviations of the averaged signal in the 100 ms prior to the stimulus. Any pulses that did not reach their individual 5 standard deviation threshold (14%, 350 of 2520 trials) were excluded from the averaging. Because latency can be altered by voluntary contraction^23^ any resting latency trials with EMG amplitude greater than 20 µV in the 100 ms prior to the stimulus (24%, 545 of 1120 trials) were also excluded from the averaging of EMG signals for resting PA and AP latency detection. Because MEP latency is more reliable than MEP amplitude^37^ we made a pragmatic decision that latency should only be calculated where at least 3 trials were available to create the averaged EMG signal. This meant that in any block of 20 latency trials the median number of trials averaged was 16 (min 3, max 20), and for LM latency all blocks were averaged from 10 trials. We ultimately obtained PA and LM active latencies for all (28) participants, AP active and AP resting for 27 participants, and PA resting for 26 participants. The averaged traces and detected latencies were plotted for visual inspection revealing 7 clear failures (of the 136 plots), due to noise or dc drift in the signal. These latencies were corrected by manually adjusting the onset to the first subsequent point clearly associated with the MEP response where the EMG signal exceeded the mean plus 5 SD of the pre-pulse EMG^7,16^. The mean plus 5 SD threshold was chosen to keep the failed detections, and hence the manual adjustments, to a minimum in order to maximize the objectivity of the extracted latency metric. We also made a further check on our automatically detected latencies (detailed and reported in the “Supplementary material”) and confirmed that they were similar to the onsets observed via visual inspection of overlay plots of all trials for each block.

For each participant, active LM latency (the indicator of D wave latency) was subtracted from both resting and active PA and AP latencies to create latency difference metrics^16^. This provided an indication of whether the CSVs evoked in each direction (PA and AP) and condition (resting or active) tended to commence with earlier (i.e. smaller values, closer to D wave latency) or later (i.e., larger values, further from D wave latency) I waves. The resulting latency difference metrics were PA-LM_ACT_, AP-LM_ACT_, PA-LM_REST_, and AP-LM_REST_.

For each participant and current direction, (excluding trials containing EMG activity as explained above) SICI was calculated by expressing the average of the conditioned MEP amplitudes as a percentage of the average unconditioned amplitudes (i.e. (conditioned amplitude/unconditioned amplitude) × 100, 100 = no inhibition, below 100 = inhibition). The resulting metrics were SICI_PA_ and SICI_AP_.

### Statistical analysis

Assumption testing, analyses, post-hoc testing, and adjustments for multiple comparison are detailed here in order to simplify the presentation of the results section that follows. Firstly, three separate preliminary comparisons were conducted to confirm our measurements were in accordance with values previously reported^16,23^. These comparisons were of the motor thresholds (AMT_PA_, AMT_AP_, AMT_LM_, SI1mV_PA_, and SI1mV_AP_), raw latencies (PA_ACTIVE_, AP_ACTIVE_, LM_ACTIVE_, PA_RESTING_, and AP_RESTING_), and latency difference scores (PA-LM_ACT_, AP-LM_ACT_, PA-LM_REST_, and AP-LM_REST_). Quantile—standardized residual plots suggested that motor thresholds, raw latencies, and latency difference scores were approximately normally distributed. However none in this series met the assumption of sphericity assessed via Mauchley’s test therefore repeated-measures ANOVA with Greenhouse–Geisser correction applied was used for comparisons. Post-hoc pair-wise comparisons with Tukey’s adjustment were then conducted (see Supplementary Material Tables S1–S3). SICI_PA_ and SICI_AP_ difference scores were normally distributed, as assessed via a Shapiro–Wilk test and density plot, therefore a paired-samples T-test was used to compare them. For these preliminary analyses effect sizes are reported using Hedge’s *g*_av_ to account for the inherent correlation between these intra-individual effects, and to facilitate any future use of these results^38^.

Scatterplots were assessed prior to the main correlational analyses and indicated no outliers or non-linear relationships were present. Linear correlations (Pearson’s) were used to test for a relationship between SICI_PA_, SICI_AP_ and each of the four latency difference metrics. We note some positive skew (a possible floor effect) was present in SICI_AP_. This may suggest a degree of caution in the interpretation of the SICI_AP_ Pearson’s coefficients. Family-wise error was controlled separately for the SICI_PA_ and SICI_AP_ comparisons with Holm-Bonferroni adjusted *p* values. Statistical analyses were performed using RStudio Version 1.4.1106.

## Results

Descriptive statistics for SICI magnitudes, MEP latencies, and motor thresholds are reported in Table 1.

**Table 1:** Descriptive statistics.

Variable	*M*	*SD*	Min	Max	*N*
**SICI (%)**					
SICI_PA_	44.10	25.78	2.55	102.90	24
SICI_AP_	26.67	23.47	1.57	69.25	26
**Raw latency (ms)**					
PA_ACTIVE_	22.46	1.55	19.10	26.30	28
AP_ACTIVE_	24.24	1.92	19.50	27.30	27
LM_ACTIVE_	20.73	1.53	17.50	23.00	28
PA_RESTING_	22.66	1.54	19.20	26.20	26
AP_RESTING_	24.00	1.82	20.20	29.10	27
**Latency difference (ms)**					
PA-LM_ACT_	1.73	0.72	0.60	3.30	28
AP-LM_ACT_	3.44	1.16	1.00	5.70	27
PA-LM_REST_	1.81	0.98	− 0.30	3.20	26
AP-LM_REST_	3.24	1.35	0.60	6.1	27
**AMT (% MSO)**					
AMT_PA_	37.82	6.69	27.00	56.00	28
AMT_AP_	49.75	7.37	36.00	61.00	28
AMT_LM_	43.46	7.77	32.00	63.00	28
**SI1mV (% MSO)**					
SI1mV_PA_	58.57	10.89	43.00	81.00	28
SI1mV_AP_	71.39	11.67	50.00	94.00	28

*SICI %* Short-Interval Intracortical Inhibition, *100%* no inhibition, *< 100%* inhibition, *PA* posterior–anterior, *AP* anterior–posterior, *LM* latero-medial; Raw Latency, ms, latencies in indicated condition; Latency Difference, ms, difference from LM latency; AMT, active motor threshold; SI1mV, stimulus intensity 1 mV; MSO, maximal stimulator output (MSO).

### Cortico-spinal excitability—the effect of current direction and stimulus intensity

Repeated-measures ANOVA indicated that there were significant differences in motor thresholds (AMT_PA_, AMT_AP_, AMT_LM_, SI1mV_PA_, and SI1mV_AP_), *F*(2.48, 67.05) = 148.82, *p* < 0.001, ω^2^ = 0.84. Post-hoc comparisons with Tukey’s adjustment indicated there were significant differences between all thresholds (see table S1 in “Supplementary Material”). The lowest was AMT_PA_ followed in increasing order by AMT_LM,_ AMT_AP_, SI1mV_PA_, and SI1mV_AP_.

### Short-interval intracortical inhibition—the effect of current direction

**Figure 2:**
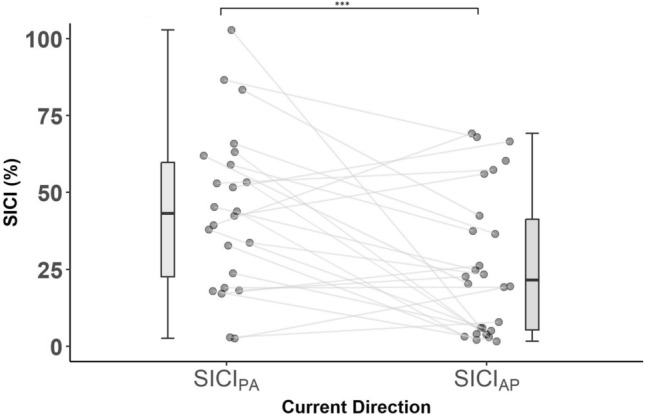
SICI in PA and AP current directions. *SICI (%)* Short-Interval Intracortical Inhibition, *100%* no inhibition, *< 100%* inhibition; Current Direction, *PA* posterior–anterior, *AP* anterior–posterior. Boxplots showing medians and IQR; ***p < 0.001.

Participants demonstrated significantly greater SICI (i.e., greater MEP suppression) when assessed with AP relative to PA current, *t*(23) = 3.02, *p* = 0.006, Hedge’s *g*_av_ = 0.68. See Fig. 2. for PA and AP SICI plot and “Supplementary Material” for discussion.

### MEP latency—indicator of I wave recruitment

Repeated-measures ANOVA indicated significant differences in raw latencies (PA_ACTIVE_, AP_ACTIVE_, LM_ACTIVE_, PA_RESTING_, AP_RESTING_), *F*(2.54, 59.22) = 69.61, *p* < 0.001, ω^2^ = 0.74. Tukey’s post-hoc comparisons (see supplementary Table S2) indicated raw latencies were significantly different across all current directions, being shortest with LM current and longest with AP current. There was no significant difference between resting and active PA, or between resting and active AP latencies, however we note these were obtained under different conditions (see “Supplementary Material” for discussion).

**Figure 3:**
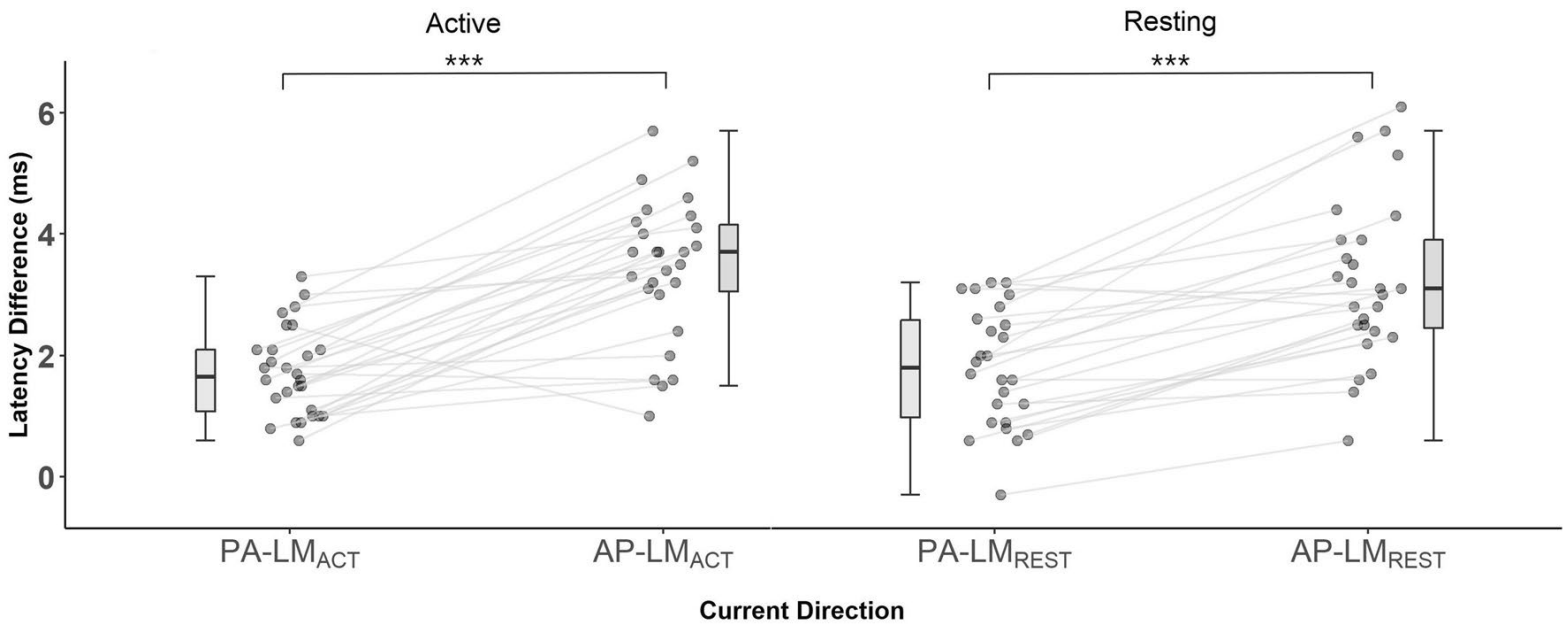
PA and AP Latency differences. Latency difference (ms), Individual PA and AP latencies subtract active LM latency; PA, posterior-anterior; AP, anterior–posterior; LM, latero-medial; Boxplots showing medians and IQR; ***p_Tukey_ < 0.001.

There were significant differences in latency difference metrics (PA-LM_ACT_, AP-LM_ACT_, PA-LM_REST_, AP-LM_REST_) *F*(2.04, 47.02) = 27.69, *p* < 0.001, ω^2^ = 0.52. Tukey’s post-hoc comparisons (see supplementary material table S3) indicated all PA measures were significantly different to all AP measures (all *p* < 0.001), but no significant differences existed between PA-LM_ACT_ and PA-LM_REST_ or AP-LM_ACT_ and AP-LM_REST_. See Fig. 3 for individual PA-LM to AP-LM latency difference plots.

### Relationship between SICI and MEP latency difference metrics

**Figure 4:**
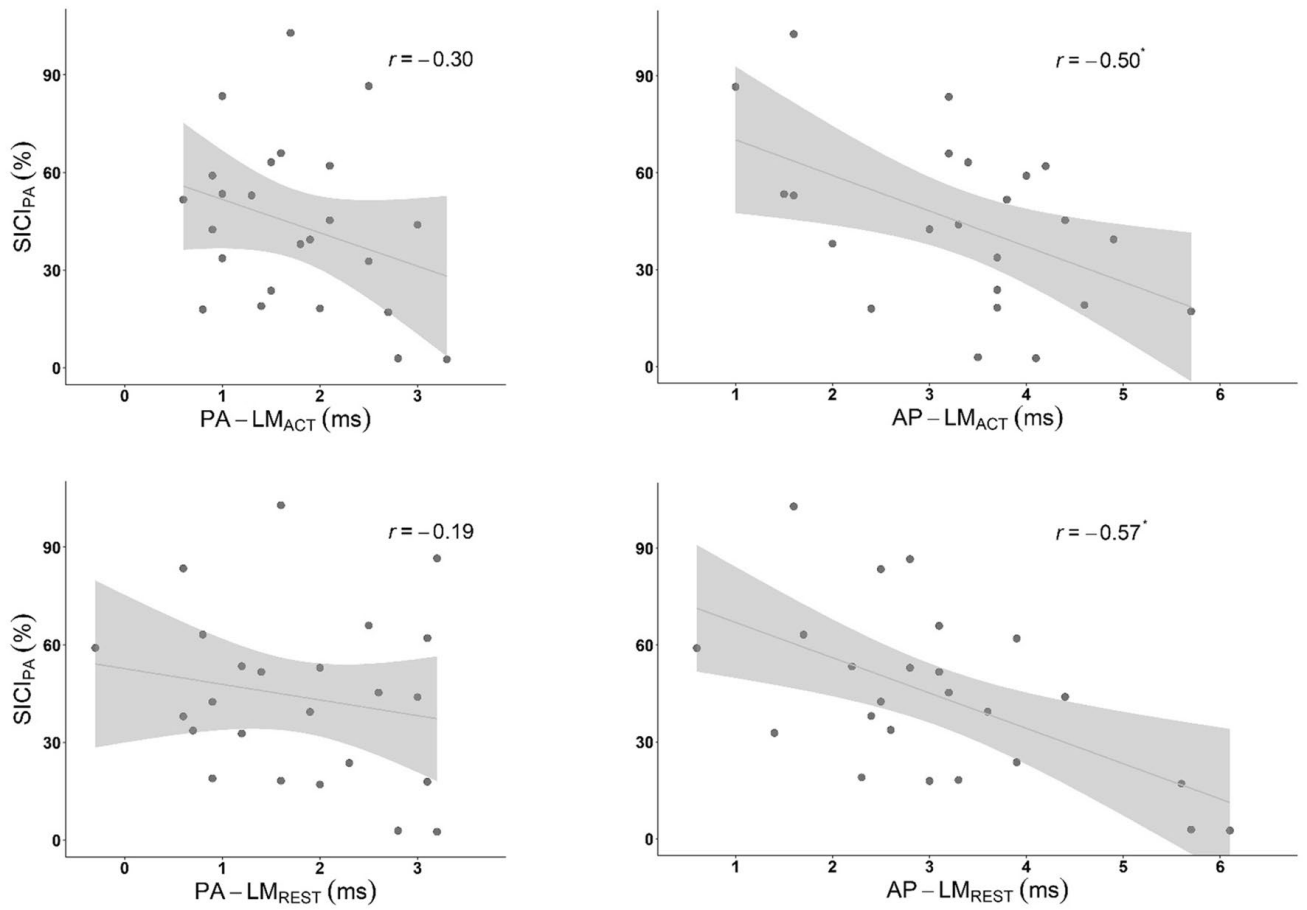
SICI_PA_ and MEP latency. SICI (%), Short-Interval Intracortical Inhibition 100% = no inhibition, < 100% = inhibition; Latency Difference (ms), Individual PA and AP latencies subtract active LM latency; PA, posterior-anterior; AP, anterior–posterior; LM, latero-medial; *r*, Pearson’s correlation coefficient; Shaded area, 95% CI; *p_adj_ < 0.05.

Greater SICI_PA_ was associated with longer AP latency differences assessed in the active, *r* = − 0.50, *r*^2^ = 0.25, *N* = 23, *p*_adj_ = 0.048 *p*_raw_ = 0.015 (two-tailed), and resting FDI, *r* = − 0.57, *r*^2^ = 0.33, *N* = 24, *p*_adj_ = 0.014, *p*_raw_ = 0.004, (two-tailed). The associations between SICI_PA_ and PA latency differences were weak and not statistically significant (active, *r* = − 0.30, *r*^2^ = 0.09, *N* = 24, *p*_adj_ = 0.303 *p*_raw_ = 0.151 (two-tailed), resting, *r* = − 0.19, *r*^2^ = 0.05, *N* = 24, *p*_adj_ = 0.379, *p*_raw_ = 0.379, (two-tailed). Scatterplots are presented in Fig. 4.

**Figure 5:**
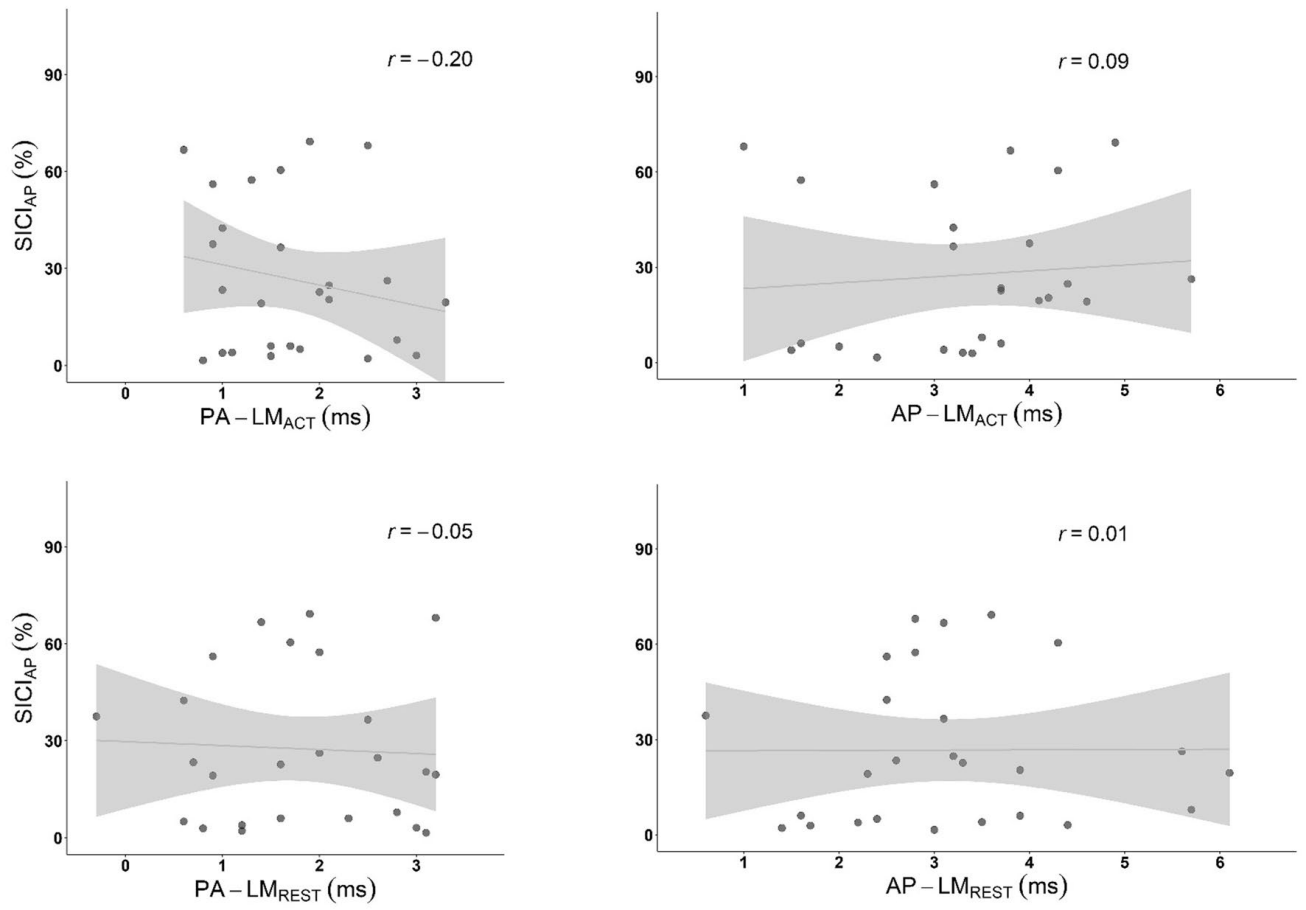
SICI_AP_ and MEP Latency. SICI (%), Short-Interval Intracortical Inhibition 100% = no inhibition, < 100% = inhibition; Latency Difference (ms), Individual PA and AP latencies subtract active LM latency; PA, posterior-anterior; AP, anterior–posterior; LM, latero-medial; *r*, Pearson’s correlation coefficient.

SICI_AP_ was not significantly associated with any latency difference: PA-LM_ACT_, *r* = − 0.20, *r*^2^ = 0.04, *N* = 26, *p*_adj_ = 1, *p*_raw_ = 0.331; PA-LM_REST_, *r* = − 0.051, *r*^2^ = 0.002, *N* = 25, *p*_adj_ = 1, *p*_raw_ = 0.807; AP-LM_ACT_, *r* = − 0.09, *r*^2^ = 0.008, *N* = 25, *p*_adj_ = 1, *p*_raw_ = 0.663; and AP-LM_REST_, *r* = − 0.005, *r*^2^ = 0.00003, *N* = 26, *p*_adj_ = 1, *p*_raw_ = 0.980. See scatterplots in Fig. 5.

## Discussion

The current study sought to further characterize the nature of the relationship between SICI and inter-individual variations in the neural populations activated by TMS (as assessed using MEP latency difference metrics). The relationship was examined, using both PA and AP current directions, by assessing MEP latency in active and resting muscle, and assessing SICI magnitude in resting muscle. Here we used conventional SICI where, because the test stimulus intensity is held constant, the composition of the test CSV is likely less variable than in the ttSICI examined previously. Our preliminary analyses indicated that mean PA-LM_ACT_ and AP-LM_ACT_ latency differences (1.7 ms and 3.4 ms respectively, see “Supplementary Material” for further discussion) were consistent with values previously used to index differences in I wave recruitment^13,16^. In line with our expectations, our results show that greater SICI_PA_ is associated with longer AP-LM MEP latency difference, explaining up to 33% of the inter-individual variability in conventional SICI. The relationship between SICI and latency difference seen here, and in previous work, has significant implications (detailed below) for the interpretation of each measure. Contrary to our expectations, SICI_AP_ was not associated with MEP latency difference.

We found that 33% of the observed individual variability in intracortical inhibition assessed with PA current was explained by AP-LM latency difference. As expected, longer AP-LM latencies were associated with greater SICI_PA_, supporting the idea that individual levels of SICI depend on individual tendency toward earlier or later I wave recruitment. Both resting and active AP-LM latencies were closely associated with SICI_PA_. To our knowledge, this is the first report of an association between AP-LM_REST_ latency and SICI_PA_, suggesting that, for our current purpose, probing MEP latency at SI1mV in resting muscle may be equally as useful as the more common 110% AMT probe used in active muscle. Our findings also support the previous account of a correlation between AP-LM_ACT_ latency and ttSICI_PA_^8^, and demonstrate here that the correlation is also apparent when the TS is held constant. While AP-LM latencies do not directly reflect the PA TS used in SICI_PA_, they are used to infer the extent of an individual’s later I wave recruitment. It seems logical that SICI’s selective inhibition of I3 and later waves, combined with SICI’s lack of impact on early I waves, could be driving the relationships we observed. We did not detect statistically significant relationships between SICI_PA_ and PA-LM latencies. Perhaps PA-LM latency is not sufficiently sensitive to the late I waves inhibited by SICI, however we note the scatterplots and coefficients appear to be in agreement with the direction of the relationship we found for AP-LM latencies.

Unlike with SICI_PA_, we observed no linear relationships between SICI_AP_ and any latency difference measure. This was in contrast to a previous report of an association between active AP-LM latency and ttSICI_AP_^7^. We acknowledge that our study may be limited by the possibility of a floor effect being present in our conventional SICI_AP_, as evidenced in the scatterplots and Fig. 4, which may have obscured any relationship between SICI_AP_ and latency difference. Alternatively, the absence of an association between SICI_AP_ and latency difference measures could be because assessing SICI with AP current avoids early I wave recruitment that would otherwise mask assessed inhibition.

Our findings of an association between conventional SICI_PA_ and AP-LM latencies represent the third report of a significant, likely neurophysiologically-driven, and arguably underappreciated relationship between SICI and MEP latency difference. Here we demonstrate for the first time that this association is present for conventional SICI, where test stimulus CSV composition is held relatively constant. Accounting for this relationship could contribute to a more individualized understanding of both GABAergic inhibitory activity and TMS preferential recruitment of distinct neural populations. The major implication here is that interpreting SICI in the context of latency difference could increase the accuracy and utility of the measure. For example, in individuals with short latency difference, who therefore have early I waves present in the CSV, the absence of SICI, i.e. no inhibition of the SICI test pulse, could mean that no later I waves were present to be inhibited, or alternatively that GABAergic activity was not apparent. In these individuals, a test pulse that reveals inhibition indicates the presence of later I waves, but the assessed SICI may only provide a diluted measure of GABAergic activity due to the presence of unaffected early I waves. However, in individuals with longer latency difference who therefore do not have early I waves present in their CSV, the extent of inhibition of the test pulse may reflect a more accurate index of GABAergic activity.

Greater precision in SICI assessment may be important in clinical investigations that report reduced SICI in a range of neurological disorders^2–4,39^. It is possible that the use of long latency difference subgroups may allow for more accurate comparisons of SICI function in clinical and healthy subjects. We also note that differences in I wave recruitment could be contributing to the SICI dysfunction identified in clinical populations. The inclusion of latency difference assessment in future SICI research could speak to this contribution, potentially increasing our understanding of the mechanisms underlying the reduced SICI observed in some clinical groups. A further implication of our findings is that conventional SICI_PA_ may provide a diluted index of GABAergic inhibitory activity. As suggested previously^40^, SICI_AP_ may deliver a more accurate assessment of intracortical inhibition by avoiding the unaffected early I wave recruitment that might mask assessed inhibition.

There are also implications for interpreting MEP latency. A growing body of research suggests that later I wave recruitment (assessed via MEP latency difference) is associated with TMS-induced neuromodulation outcomes, and with learning^16,41–44^, but because latency can only reflect the first component of the CSV, short latencies cannot speak to the presence of later I waves. In individuals with short latency difference the presence of SICI could indicate that later I waves are also present in their CSVs. We suggest that including SICI assessment in future research could, at the individual level, facilitate a more detailed understanding of how preferential TMS recruitment of distinct neural populations impacts TMS neuromodulation outcomes.

Our study used single and paired pulse TMS to examine the relationship between MEP latency difference and SICI assessed using PA and AP current. Latency difference was used to indicate whether individual motor response to TMS tended to commence with earlier or later I waves. We found that a significant proportion of the observed variability in PA SICI magnitude can be accounted for by MEP latency difference, reflecting individual differences in the neural populations preferentially activated by TMS. However, MEP latency difference did not account for the variability we observed in AP SICI. We suggest that interpreting SICI measures in the context of individual I wave recruitment patterns will contribute to more precise assessment of GABAergic intracortical inhibition, that AP SICI could more accurately reflect inhibitory processes, and that accounting for SICI could enhance our understanding of the relationship between MEP latency difference and TMS neuromodulation outcomes.

## Supplementary Information

Supplementary Information.

## Data Availability

The datasets generated during and/or analysed during the current study are available from the corresponding author on reasonable request.
