# Supplementary material for: Assessment of cortical inhibition depends on inter individual differences in the excitatory neural populations activated by transcranial magnetic stimulation
Source: Sci Rep. 2022 Jun 15;12:9923. doi: 10.1038/s41598-022-14271-1 (PMC9200840; doi:10.1038/s41598-022-14271-1)
Supplement: Supplementary file 1 — Supplementary Information. [file 41598_2022_14271_MOESM1_ESM.docx]

**Figure S1.** Corresponding TMS coil orientations, MEPs, and diagrammatic CSVs


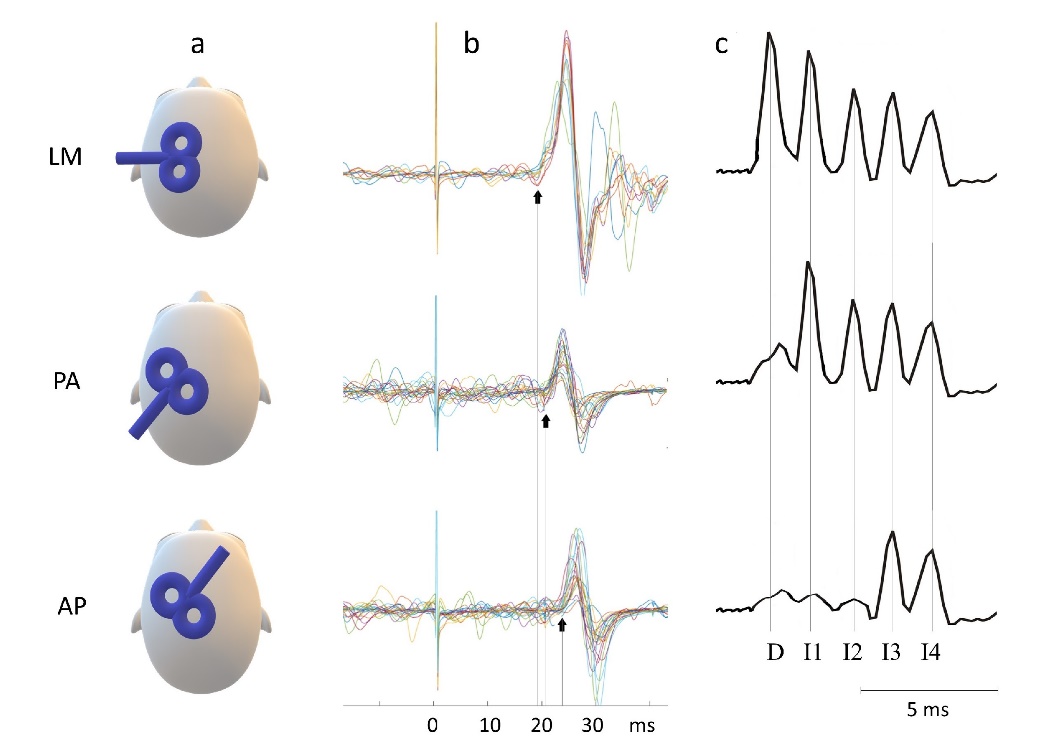


**a**, representation of TMS coil orientations and cortical current directions; LM, latero-medial; PA, posterior-anterior; AP, anterior-posterior. **b**, Corresponding MEP latencies from one participant, x-axis (ms) showing stimulus at zero ms, black arrow at approximate MEP onset. **c**, Illustrative depiction of likely corresponding CSV composition.

**Figure S2.** Effect of SICI on MEPs (**a**) and the CSV (**b**)


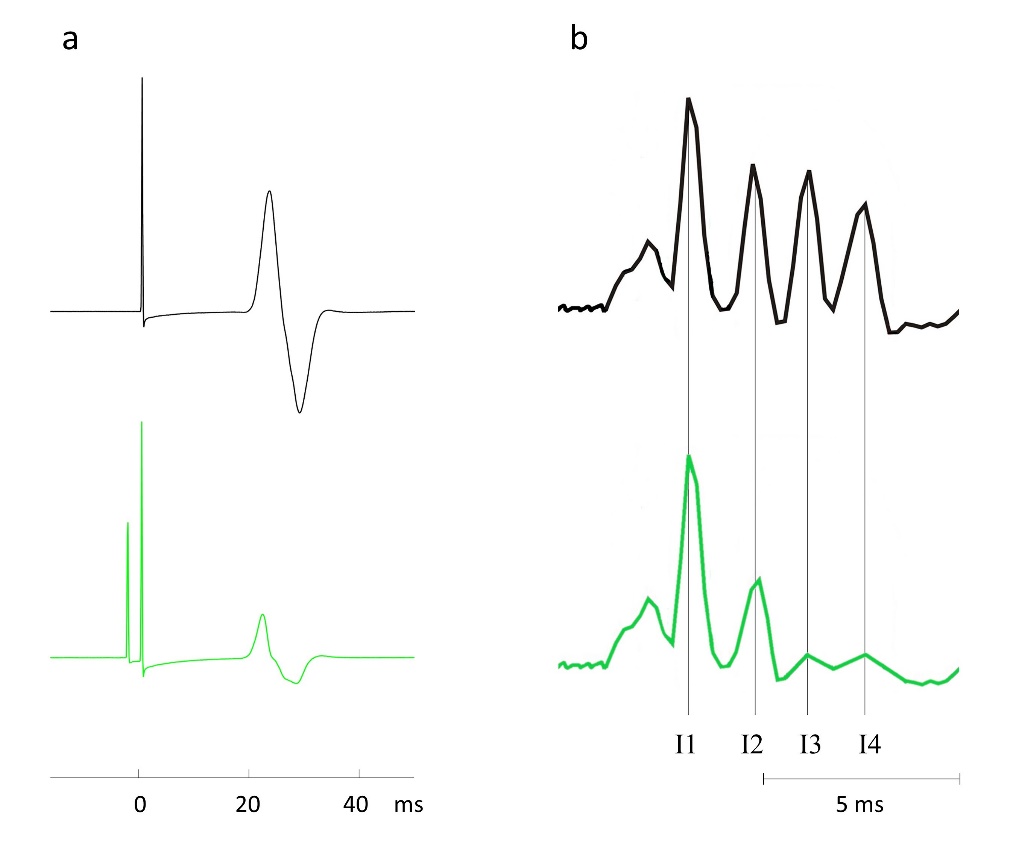


Black lines represent response to test stimuli alone, green lines represent SICI conditioned response. **a**, Average of 20 MEP responses in one participant. Test stimulus at zero ms (black) and preceded by lower intensity conditioning stimulus (green) showing reduction in peak to peak motor-evoked potential amplitude. **b**, Illustrative depiction of likely corresponding cortico-spinal volley composition, green trace showing I1 wave unaffected by SICI while later I waves are inhibited.

Table S1. Post-hoc Comparison of Motor Thresholds

| Comparison | | M_Diff_ (% MSO) | SE | df | t | p_Tukey_ | Hedge’s g_av_ |
| --- | --- | --- | --- | --- | --- | --- | --- |
| AMT_PA_ | AMT_AP_ | -11.93 | 1.14 | 27.00 | -10.48 | < .001 | 1.65 |
|  | AMT_LM_ | -5.64 | 0.89 | 27.00 | -6.31 | < .001 | 0.76 |
|  | SI1mV_PA_ | -20.75 | 1.57 | 27.00 | -13.19 | < .001 | 2.23 |
|  | SI1mV_AP_ | -33.57 | 1.86 | 27.00 | -18.01 | < .001 | 3.43 |
| AMT_AP_ | AMT_LM_ | 6.29 | 1.53 | 27.00 | 4.11 | 0.003 | 0.81 |
|  | SI1mV_PA_ | -8.82 | 1.58 | 27.00 | -5.57 | < .001 | 0.92 |
|  | SI1mV_AP_ | -21.64 | 1.42 | 27.00 | -15.22 | < .001 | 2.62 |
| AMT_LM_ | SI1mV_PA_ | -15.11 | 1.72 | 27.00 | -8.76 | < .001 | 1.55 |
|  | SI1mV_AP_ | -27.93 | 1.97 | 27.00 | -14.14 | < .001 | 2.87 |
| SI1mV_PA_ | SI1mV_AP_ | -12.82 | 1.31 | 27.00 | -9.76 | < .001 | 1.44 |

Note. Comparison, pair-wise comparison; M_Diff_, mean difference of pair; MSO, maximal stimulator output.

Table S2. Post-hoc Comparison of Raw Latencies

| Comparison | | M_Diff_ (ms) | SE | df | t | p_Tukey_ | Hedge’s g_av_ |
| --- | --- | --- | --- | --- | --- | --- | --- |
| PA_ACTIVE_ | AP_ACTIVE_ | -1.66 | 0.26 | 23.00 | -6.50 | < .001 | .99 |
|  | LM_ACTIVE_ | 1.71 | 0.15 | 23.00 | 11.27 | < .001 | 1.08 |
|  | PA_RESTING_ | -0.10 | 0.16 | 23.00 | -0.62 | 0.969 | 0.13 |
|  | AP_RESTING_ | -1.51 | 0.19 | 23.00 | -7.94 | < .001 | 0.88 |
| AP_ACTIVE_ | LM_ACTIVE_ | 3.37 | 0.24 | 23.00 | 14.04 | < .001 | 1.96 |
|  | PA_RESTING_ | 1.56 | 0.32 | 300 | 4.88 | < .001 | 0.88 |
|  | AP_RESTING_ | 0.15 | 0.27 | 23.00 | 0.55 | 0.981 | 0.12 |
| LM_ACTIVE_ | PA_RESTING_ | -1.81 | 0.20 | 23.00 | -8.87 | < .001 | 1.22 |
|  | AP_RESTING_ | -3.22 | 0.27 | 23.00 | -11.96 | < .001 | 1.88 |
| PA_RESTING_ | AP_RESTING_ | -1.41 | 0.20 | 23.00 | -7.07 | < .001 | 0.77 |

Note. Comparison, pair-wise comparison; M_Diff_, mean difference of pair in milliseconds.

Table S3. Post-hoc Comparison of Latency Difference

| Comparison | | M_Diff_ (ms) | SE | df | t | p_Tukey_ | Hedge’s g_av_ |
| --- | --- | --- | --- | --- | --- | --- | --- |
| PA-LM_ACT_ | AP-LM_ACT_ | -1.65 | 0.26 | 23.00 | -6.50 | < .001 | 1.71 |
|  | PA-LM_REST_ | 0.10 | 0.16 | 23.00 | -0.63 | 0.922 | 0.08 |
|  | AP-LM_REST_ | -1.51 | 0.19 | 23.00 | -7.94 | < .001 | 1.35 |
| AP-LM_ACT_ | PA-LM_REST_ | 1.56 | 0.32 | 23.00 | 4.88 | < .001 | 1.47 |
|  | AP-LM_REST_ | 0.15 | 0.27 | 23.00 | 0.55 | 0.945 | 0.15 |
| PA-LM_REST_ | AP-LM_REST_ | -1.41 | 0.20 | 23.00 | -7.07 | < .001 | 1.17 |

Note. Comparison, pair-wise comparison; M_Diff_, mean difference of pair in milliseconds.

Latency and I-wave Recruitment

At a group level, mean PA-LM_ACT_ and AP-LM_ACT_ latency differences were 1.7ms and 3.4 ms respectively, consistent with values previously used to index differences in I wave recruitment. We also assessed latency in resting muscle because we thought the composition of the CSV recruited by the test pulse used to probe conventional SICI could affect SICI magnitude. PA-LM_REST_ and AP-LM_REST_ latency differences, were approximately 1.8ms and 3.2 ms respectively. Interestingly, resting latencies were similar to their active counterparts. Perhaps because (a) our threshold for latency detection was reduced by the lower EMG variability in resting muscle; and (b) the higher stimulus intensity used in resting trials may have shortened MEP latency.

Current Direction and SICI

Consistent with previous findings, SICI assessed with AP current was significantly greater than when assessed with PA current. The lower levels of SICI we observed using PA current may be because early I waves are more often present in CSVs elicited by PA stimulation. Here, since early I waves are unaffected by SICI conditioning, they contribute to test MEP amplitude and reduce the assessed inhibition. The greater inhibition we observed with AP directed current may be because AP CSVs less frequently contain early I waves unaffected by SICI, but instead tend to commence with the later I waves that are selectively reduced by SICI. Alternatively, it may be possible that an AP directed conditioning stimulus activates different SICI circuits that produce greater inhibition.

Additional check of automatically detected latencies

As an additional check, the responses for each block were overlaid and the earliest onset was manually identified by two independent researchers (AC, BS). These visual latencies compared well with each other (ICC 0.927, 95% CI 0.890 < ICC < 0.952, p < .001) and to the automatically identified latencies (ICC 0.920, 95% CI 0.879 < ICC < 0.948, p < .001, and ICC 0.832, 95% CI 0.753 < ICC < 0.888, p < .001). Due to their greater objectivity, MEP latencies identified by the automated procedure were used for all analyses.
